# Endovascular and Open Surgical Repair of Intact Abdominal Aortic Aneurysms: Outcomes, Treatment Trends and Early Mortality Risk Factors in a Single-Centre Study over 18 Years †

**DOI:** 10.3390/jcm15041353

**Published:** 2026-02-09

**Authors:** Maria Del Pilar Ortega Carrillo, Oksana Heiser, Shamsun Naher, Benedikt Reutersberg, Albert Busch, Matthias Trenner, Michael Kallmayer, Christoph Knappich

**Affiliations:** 1 Department of Vascular and Endovascular Surgery, Munich Aortic Centre, TUM University Hospital, Klinikum Rechts der Isar, Technical University of Munich, 81675 Munich, Germany; 2 Department of Vascular Surgery, University Hospital Zurich, 8091 Zurich, Switzerland; 3 Division of Vascular and Endovascular Surgery, Department for Visceral, Thoracic and Vascular Surgery, Medical Faculty Carl Gustav Carus and University Hospital, Technical University Dresden, 01307 Dresden, Germany; 4 Division of Vascular Medicine, St. Josefs Hospital, 65189 Wiesbaden, Germany

**Keywords:** aortic aneurysm, abdominal, EVAR, vascular grafting, survival analysis, OSR, risk factors, perioperative mortality

## Abstract

**Background**: Endovascular aortic repair (EVAR) has become the standard approach for intact abdominal aortic aneurysm (iAAA) repair in recent decades. This retrospective single-centre study analyzed real-world treatment trends, early outcomes, and risk factors for in-hospital mortality following EVAR and open surgical repair (OSR) in patients treated for iAAA from 2005 to 2022. **Methods**: In-hospital mortality was the primary outcome. Univariate and multivariable logistic regression analyses were performed. Logistic and logarithmic regression models were established to assess temporal changes. **Results**: In total, 1237 patients (90% male, mean age 72 years) were included, comprising 863 EVAR and 374 OSR procedures. In-hospital mortality was lower after EVAR than OSR (0.93% vs. 3.48%; *p* = 0.001). After risk adjustment, higher age (adjusted odds ratio [OR] 2.39, 95% confidence interval [CI] 1.26–4.78; *p* = 0.007) and chronic obstructive pulmonary disease (OR 4.43, 95% CI 1.82–10.8; *p* = 0.001) were associated with higher in-hospital mortality. Conversely, EVAR (vs. OSR; OR 0.16, 95% CI 0.06–0.43; *p* < 0.001) was associated with a lower hospital mortality risk. Throughout the study, in-hospital mortality rates for EVAR and OSR remained stable. With a median follow-up of 21 months, OSR-treated patients presented prolonged survival during follow-up (*p* = 0.021). **Conclusions**: EVAR represents a reliable alternative for elderly patients with multiple comorbidities considered unfit for OSR. Although EVAR was associated with lower in-hospital mortality, survival was lower during the 5-year follow-up compared to OSR.

## 1. Introduction

Since its introduction in 1991, endovascular aortic repair (EVAR) has become established as an alternative treatment for patients with abdominal aortic aneurysms (AAAs). Patients with a higher comorbidity burden face an increased mortality risk during open surgical repair (OSR) [1]. Over the past three decades, EVAR has continued to evolve, including advances in stent graft technologies for challenging anatomies as well as percutaneous implantation of devices [2,3,4]. The most evident advantages of EVAR comprise reduced hospital and intensive care unit (ICU) stays and fewer surgical complications [1,5]. As a result, EVAR can be performed safely and has become the preferred treatment option for patients deemed unfit for OSR [6,7,8].

Findings from randomized controlled trials (RCTs) comparing EVAR and OSR are consistent regarding the early benefit of EVAR in terms of 30-day mortality and morbidity [9,10,11]. As the survival advantage diminishes 3 years after the index procedure, the need for reintervention after EVAR increases [12,13,14]. Therefore, the European Society for Vascular Surgery (ESVS) guidelines encourage the use of EVAR only if the anatomy is suitable. At the same time, OSR should be considered the preferred treatment modality for patients with a long life expectancy [15]. However, data from international registry studies portray a behaviour that differs from these recommendations: over the past few decades, the overall use of EVAR has increased regardless of patients’ age [16].

This retrospective study evaluates in-hospital and 5-year follow-up outcomes, risk factors, and treatment trends concerning EVAR and OSR for non-ruptured (intact) AAA (iAAA) in a single-centre experience over 18 years.

## 2. Materials and Methods

### 2.1. Study Design

This study comprises a retrospective descriptive analysis of patients presenting with iAAA and treated between 1 January 2005 and 31 December 2022. Patients were identified from the prospectively maintained aortic database of the Klinikum rechts der Isar (TUM University hospital) of the Technical University of Munich (TUM). The study adheres to the STROBE guidelines [17] and reporting standards for EVAR [18,19]. The ethics committee of the TUM University Hospital approved the data collection under reference number 2025-177-S-NP.

### 2.2. Patients and Procedures

Patients undergoing EVAR or OSR for iAAA were included. This definition encompassed juxtarenal (neck length < 10 mm) and infrarenal (neck length > 10 mm) AAAs with or without associated iliac aneurysms. Suitability for EVAR was based on anatomic criteria; patients with a life expectancy of ≥10 years were offered OSR if declared fit. Patients presenting with abdominal or lumbar pain and, by definition, with a symptomatic aneurysm, were also included in the study. The iAAA diagnosis was confirmed by computed tomography angiography (CTA). All procedures were performed according to the standard operating procedures at our aortic centre and under general anaesthesia. Access for EVAR was obtained via femoral artery cut-down or percutaneously using ultrasound. The main body introduction site was left to the discretion of the surgeon. Stent grafts were predominantly aorto-biiliacal; however, in some cases, stent graft types varied based on anatomy.

**Figure 1:**
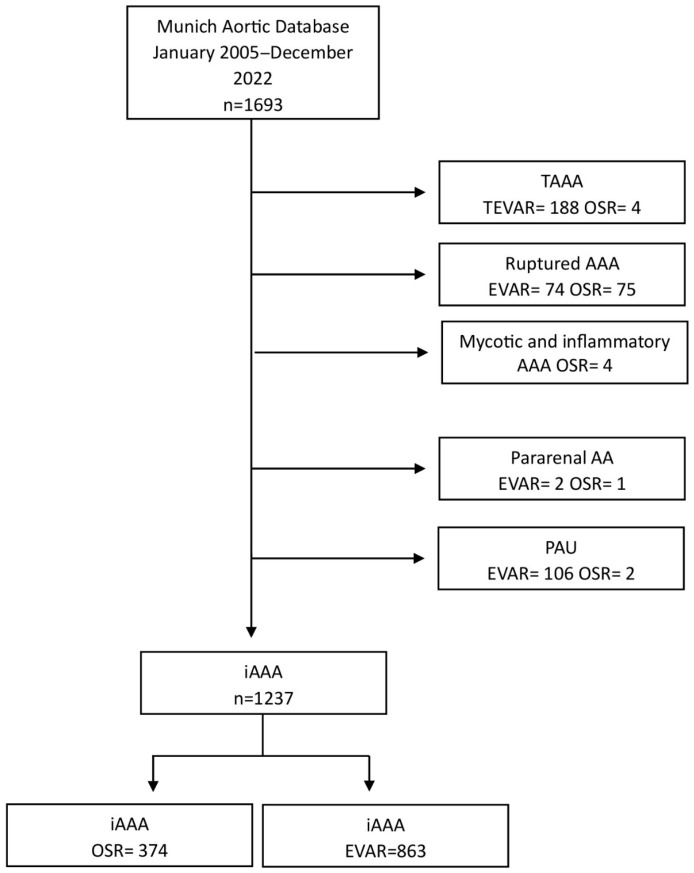
Flowchart of patient selection from the Munich Aortic Database from January 2005 to December 2022. TAAA = thoracoabdominal aortic aneurysm, AAA = abdominal aortic aneurysm, TEVAR = thoracic endovascular aortic repair, OSR = open surgical repair, EVAR = endovascular aortic repair, iAAA = intact abdominal aortic aneurysm, PAU = penetrating aortic ulcer.

Open surgical repair was performed through median laparotomy or a left retroperitoneal approach. Reconstruction was performed with tubes, aorto-biiliacal or aorto-bifemoral grafts. Furthermore, infrarenal, suprarenal, supramesenteric or supraceliac clamping was utilized depending on aneurysm’s morphology and anatomy. Patients with a penetrating aortic ulcer; a pararenal or thoracoabdominal aortic aneurysm; and mycotic, inflammatory, or ruptured AAAs were excluded from the analysis. Patients who had previously undergone AAA repair were also excluded. The patient flowchart is shown in Figure 1.

### 2.3. Data Collection

Patients’ clinical data including anatomic characteristics; preoperative, operative and postoperative characteristics; and other hospitalization data were obtained from electronic medical records. After pseudonymization, the data were prospectively entered into an electronic data system. Follow-up data were obtained from the electronic records of our outpatient department. The follow-up algorithm consisted of follow-up visits at 1, 6, and 12 months after the index procedure. Thereafter, follow-up was conducted annually. If significant endoleaks or sac enlargement after EVAR and stenosis or aneurysm of the anastomosis after OSR were observed, follow-up visits and imaging outside the algorithm could be scheduled. The follow-up imaging algorithm changed over the observation period. A duplex ultrasound was conducted at every visit. In the early study observation period, CTA scans were performed at 1 month, 1 year and 5 years after the index procedure. In the later observation period, contrast-enhanced ultrasound replaced the CTA scan 1 month after the procedure to reduce patients’ radiation exposure. The follow-up data on reinterventions or death were complemented by contacting the general practitioner or family members.

### 2.4. Outcomes and Follow-Up

The primary outcome was defined as death after the index procedure during hospitalization or in-hospital mortality. Secondary outcomes included risk factors for In-hospital mortality, survival during follow-up, and treatment trends during the study period. In addition, a detailed descriptive analysis was conducted regarding perioperative outcomes, such as lengths of hospital and ICU stays, and surgical and medical complications. Complications were categorized according to the Clavien–Dindo classification [20]. Surgical complications were classified as minor and major complications. Medical complications included respiratory, renal, cardiac, gastrointestinal, and neurological complications. Survival rates are based on the follow-up visits 30 days, 6 months and up to 5 years after the index procedure. Patients who did not complete follow-up and those who died, but whose exact date of death could not be ascertained, were censored at the last visit.

### 2.5. Analyses

Clinical variables were evaluated at different time intervals. Categorical variables were presented as absolute numbers and percentages. Continuous data were presented as median and interquartile range if not normally distributed. Normally distributed interval-scaled data were presented as mean and standard deviation. Chi-squared and Fisher’s *t*-test were used to determine differences in nominally and interval-scaled data between EVAR and OSR. Mood’s median test was used to compare the medians of two or more independent groups of numeric data, and the comparison of means was by *t*-test. Treatment trends were analysed separately for EVAR and OSR using linear, logistic and negative binomial regression modelling. Patients were divided into three groups according to the treatment date (2005–2010, 2011–2016 and 2017–2022). The first group was defined as a reference for overall time changes. The results are presented as differences per year (∆ per year). Univariate analyses were used to determine risk factors clinically associated with in-hospital mortality. A multivariate regression model was applied to adjust for potential confounders. Variable selection based on established evidence and clinical relevance. Due to the limited number of events (*n* = 21), Firth’s penalized logistic regression was applied to reduce bias and obtain reliable parameter estimates. In our analysis, the EPV was approximately 5, which is considered acceptable for Firth’s logistic regression. After calculating McFadden’s R-squared (0.54) for the logistic regression model, the final model fit our data. Survival was analyzed using the Kaplan–Meier method. Since the patients’ survival data after the index procedure were proportional, a stepwise Cox proportional regression model for the assessment of survival was applied. The multivariate analysis model was fit, since a normal data distribution could be demonstrated using a Q–Q plot (Supplemental Figure S1). *p*-values < 0.05 were considered statistically significant. Statistical analyses were performed using RStudio, version 4.4.2 (R Foundation, Vienna, Austria). Four patients in the endovascular group were converted to open surgery during the procedure due to failure of stent graft implantation or acute stent graft thrombosis. These patients were considered based on the intention to treat. Missing patient demographic and comorbidity data were imputed using the multivariate imputation by chained equations (MICE) method for multivariate analysis.

## 3. Results

### 3.1. Baseline Characteristics

Overall, 1237 patients were included in our analyses, of whom 374 patients underwent OSR (Table 1). With a mean age of 72 years in the entire cohort, patients who underwent EVAR were older than OSR patients (74 vs. 68 years; *p* < 0.001). While EVAR patients had a higher rate of infrarenal AAAs (88% vs. 76%; *p* < 0.001), those in the OSR group presented significantly higher percentages of juxtarenal aneurysms (24% vs. 12%; *p* < 0.001) and concomitant iliac aneurysms (15% vs. 7.4%; *p* < 0.001). Patients treated with EVAR had higher rates of cardiovascular comorbidities. Additionally, these patients exhibited a higher mean body mass index (BMI), increased rates of active malignant disease or cancer treatment in the past 10 years, and a greater percentage of preoperative American Society of Anesthesiologists (ASA) class III (*p* = 0.005). The OSR-treated patients had higher smoking and COPD rates as well as a higher percentage of patients with renal insufficiency.

### 3.2. Intraoperative Characteristics

The most frequently used surgical access for OSR was left retroperitoneal (58%), with the median duration of surgery at 210 min. A tube graft was used in around 60% of the repairs. Infrarenal clamping was performed in 73% of cases, and 16% required suprarenal clamping (Supplementary Table S1). In the EVAR cohort, percutaneous access was applied in 44% of cases, with a median procedure time of 123 min. The rate of type II, type I and type III endoleaks at discharge was 19%, 5.21% and 2.20%, respectively (Supplementary Table S2).

### 3.3. Perioperative Outcomes

After EVAR treatment (Table 2), patients stayed in the hospital for a shorter time than after OSR (7 vs. 10 days; *p* < 0.001). The mean ICU stay was shorter following EVAR compared to OSR (0 vs. 1 day; *p* < 0.001). Perioperative mortality was significantly lower after EVAR than after OSR (0.93% vs. 3.48%; *p* = 0.001). The specific causes of in-hospital mortality are provided in Supplementary Table S3. The incidence of medical complications was lower in the EVAR cohort (10% vs. 35%; *p* < 0.001). Additionally, lower rates of surgical complications during hospitalization were shown (10% vs. 16%; *p* = 0.008). The types of surgical complications are listed in Supplementary Table S4.

### 3.4. Time Trends

Over the observation period (Table 3), no significant changes were observed for EVAR in terms of the maximum AAA diameter in 2011–2016 and 2017–2022 compared to the baseline period of 2005–2010 (∆ −0.05; *p* = 0.44). The mean patient age showed a non-significant upward trend from 2005 to 2022 (∆ 0.08; *p* = 0.17). Perioperative mortality remained stable throughout the study period (∆ 0.08; *p* = 0.35). The median procedure time decreased significantly over time (∆ −1.97; *p* = 0.002). The use of percutaneous access (∆ 0.64; *p* < 0.001) and fenestrated endografts (∆ 0.15; *p* < 0.001) increased over the later years.

In the OSR cohort (Table 4), no relevant change in patient age (∆ 0.04; *p* = 0.61), maximum AAA diameter (∆ −0.003; *p* = 0.98), and perioperative mortality (∆ 0.01; *p* = 0.79) was observed compared to the baseline cohort. Furthermore, the number of medical (∆ 0.02; *p* = 0.34) and surgical complications (∆ 0.04; *p* = 0.17) remained constant. The number of days of hospital stay (∆ 0.02; *p* < 0.001), the procedure time (∆ 3.29; *p* < 0.001), the use of suprarenal clamping (∆ 0.06; *p* = 0.037), and left retroperitoneal access (∆ 0.10; *p* < 0.001) increased in the later periods.

**Figure 2:**
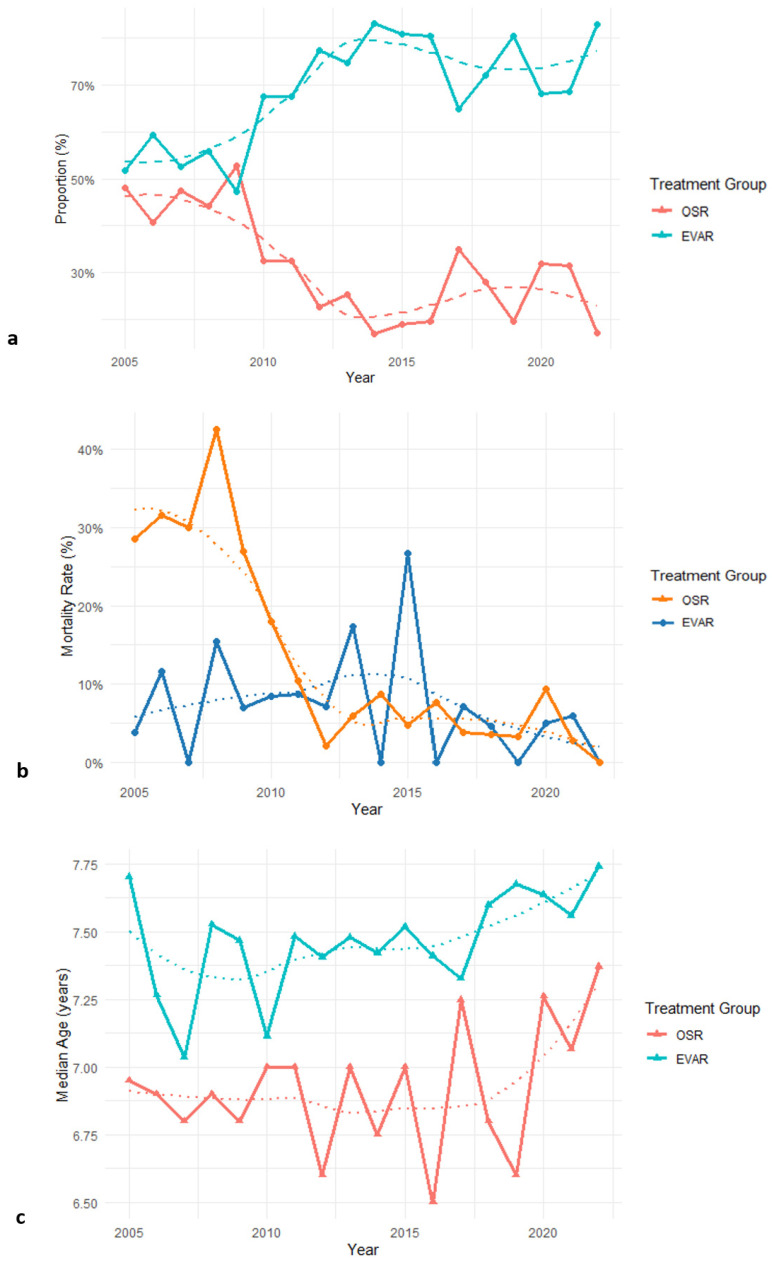
Temporal trends for EVAR and OSR between 2005–2022. (**a**) Proportion of EVAR vs. OSR over time. (**b**) Annual mortality by treatment group. (**c**) Median patient Age by treatment group.

The number of EVAR procedures presented an upward trend from 2005 to 2022, while the use of OSR decreased. This trend is illustrated in Figure 2. Additionally, the data indicated an overall lower hospital mortality for EVAR, as shown in Figure 2b. Both treatment groups exhibited yearly variability in mortality rates with non-significant trends (EVAR ∆ 0.08; *p* = 0.35 vs. OSR ∆ 0.01; *p* = 0.79). The median age of patients in the EVAR group was higher, and the age of patients in both groups presented a non-significant upward trend over the years (EVAR ∆ 0.08; *p* = 0.17 vs. OSR ∆ 0.04, *p* = 0.61; Figure 2c).

### 3.5. Univariate Analyses

Univariate analysis revealed that the presence of COPD (odds ratio [OR] 3.92, 95% confidence interval [CI] 1.60–9.50; *p* = 0.003) and ASA class IV (OR 7.87, 95% CI 2.38–26.0; *p* = 0.001) were associated with higher hospital mortality. Conversely, implementation of EVAR (OR 0.26, 95% CI 0.10–0.63; *p* = 0.001) and infrarenal AAAs (OR 0.09, 95% CI 0.03–0.21; *p* < 0.001) were associated with lower in-hospital mortality (Table 5).

### 3.6. Multivariable Regression Analysis

**Figure 3:**
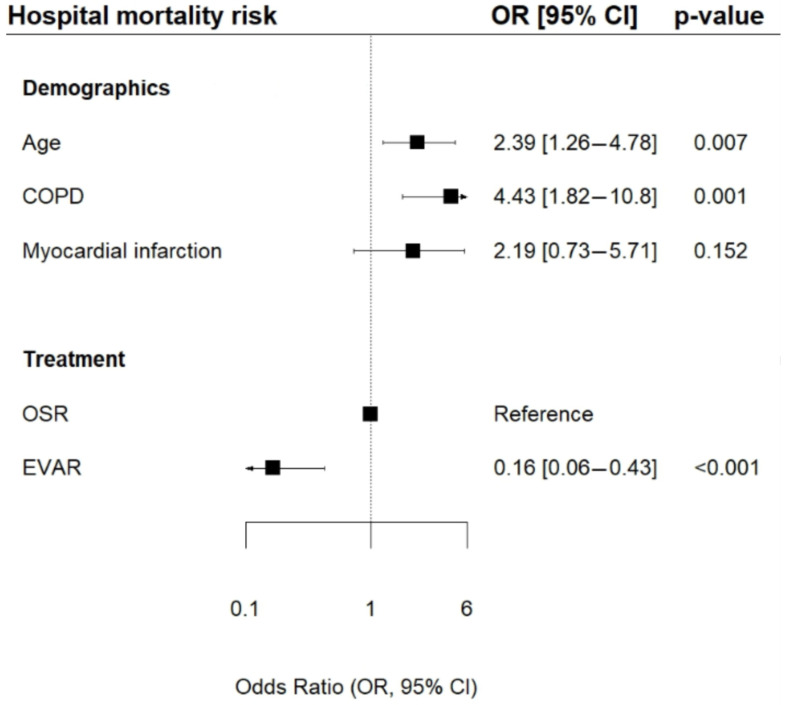
Multivariate regression analysis: Risk of in-hospital mortality of intact abdominal aneurysm. OR = adjusted odds ratio, CI = confidence interval, COPD = chronic obstructive disease, EVAR = endovascular aortic repair, OSR = open surgical repair.

After adjusting for confounders, we applied a Firth’s penalized logistic regression model (Figure 3). The presence of COPD (OR 4.43, 95% CI 1.82–10.8; *p* = 0.001) and higher age (OR 2.39, 95% CI 1.26–4.78; *p* = 0.007) were associated with higher in-hospital mortality. Moreover, a significantly lower risk for hospital mortality after EVAR compared to OSR was observed (OR 0.16, 95% CI 0.06–0.43; *p* < 0.001).

### 3.7. Survival and Follow-Up

**Figure 4:**
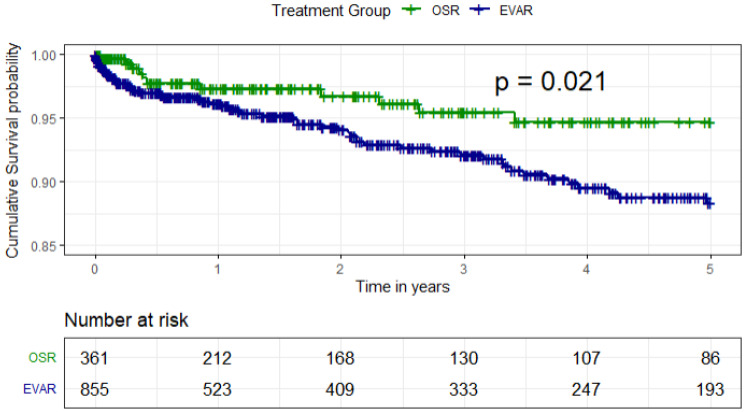
Kaplan–Meier curve of cumulative survival for EVAR and OSR over 5 years. EVAR = endovascular aortic repair, OSR = open surgical repair.

**Figure 5:**
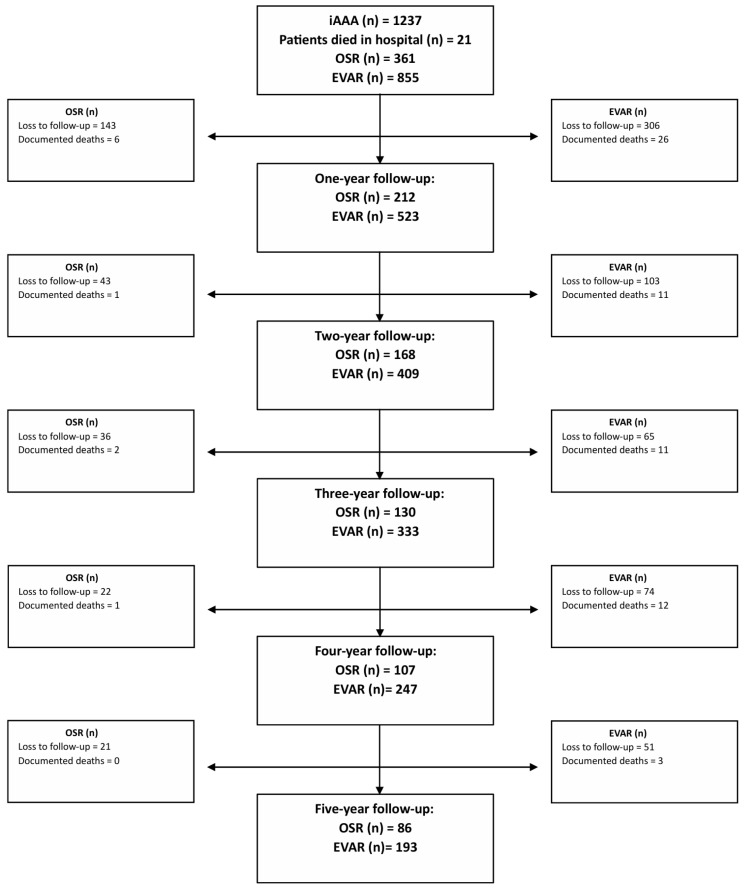
Flowchart of patients during follow-up. EVAR = endovascular aortic repair, OSR = open surgical repair.

The cumulative probability of 5-year survival was higher for patients receiving OSR compared to EVAR (*p* = 0.021; Figure 4). After conducting Cox proportional regression, this survival probability persisted between the two treatment groups (*p* = 0.042; Supplementary Figure S2). Conversely, higher age and cancer history were associated with a lower cumulative probability of survival after the index procedure (Supplementary Figure S2). The patients remaining at risk after treatment are depicted in Figure 5.

**Figure 6:**
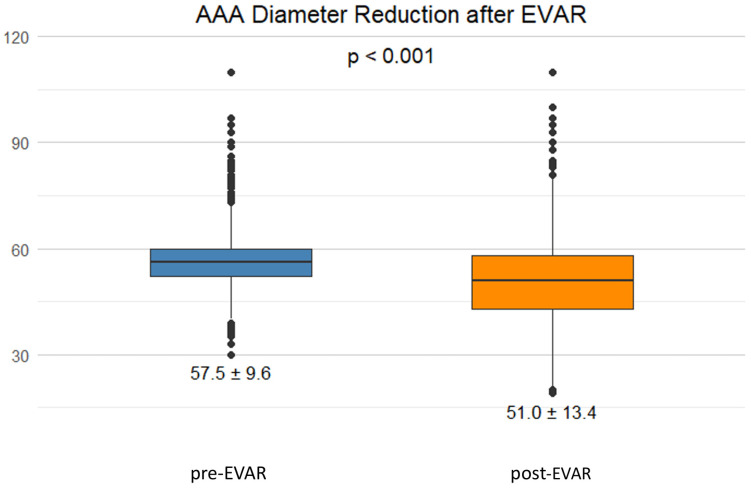
Boxplot displaying a significant reduction in aneurysm diameter after EVAR (in mm; mean, SD). EVAR = endovascular aneurysm repair.

Of the EVAR-treated patients, 110 (13%) were lost to follow-up directly after discharge. With a median follow-up of 21 months, 333 patients (38%) underwent contrast-enhanced ultrasound and 281 patients (32%) CTA. Around 15% of patients in the EVAR cohort had a non-enhanced ultrasound during their follow-up visit (Supplementary Table S6). We observed a reduction in the maximum aneurysm diameter at the last follow-up (*p* < 0.001; Figure 6). Among the OSR-treated patients, the majority (*n* = 247) underwent ultrasound control after treatment. Around 14% (*n* = 51) of the OSR patients underwent CTA. The main causes of death during follow-up are displayed in Supplementary Table S5.

## 4. Discussion

This 18-year single-centre study demonstrated lower in-hospital mortality rates among patients treated with EVAR compared to patients undergoing OSR (0.93% vs. 3.48%) in routine clinical practice. Additionally, lower rates of medical and surgical complications were observed after EVAR. These favorable perioperative outcomes after EVAR compared to OSR align with previously published findings [5,9]. For instance, a meta-analysis of seven RCTs comparing EVAR to ORS reported in-hospital mortality rates of 1.4% and 4.5%, respectively [9]. While the relative difference in mortality rates is consistent with the present single-centre study, the absolute values in the meta-analysis were higher for both procedures as compared to our study.

One explanation for the lower perioperative mortality seen with EVAR in our study might be that the meta-analysis included patients treated in the late 1990s and early 2000s, and devices and implantation techniques might have improved since then. Similarly, the discrepancy in mortality rates after OSR may reflect contemporary trends in treatment selection, as older or more comorbid patients are increasingly managed with EVAR. After risk adjustment, the meta-analysis showed EVAR to be independently associated with lower in-hospital mortality than OSR. On the contrary, despite our extended multivariate logistic regression model demonstrating an independent effect of EVAR on in-hospital mortality, the model was overfitted. The low number of deaths and the spread of the 95% CI prevent generalization of these findings.

Additionally, factors such as older age, COPD and a history of myocardial infarction were independently associated with higher in-hospital mortality. The significantly higher rates of juxtarenal aneurysms and COPD in patients who had undergone OSR—apart from the greater invasiveness of the procedure—could explain the higher in-hospital mortality rates. A recent registry study presented similar results. The authors categorized patients into low-, moderate-, and high-risk groups while comparing hospital mortality rates for EVAR and OSR. COPD, higher age, and renal insufficiency were identified as characteristics of high-risk patients. Among these patients, a higher hospital mortality was observed in those treated with OSR [21]. Other meta-analyses and real-life experiences associate older age with higher hospital mortality rates after iAAA repair. The risk of hospital mortality increased following OSR [22,23]. These findings underscore the importance of considering comorbidities and adhering to guideline recommendations in treatment and patient selection.

In contrast to reports of a slight reduction in the in-hospital mortality associated with both EVAR and OSR in recent years, our data show stable in-hospital mortality rates between 2005 and 2022 [1,24]. These findings underscore the fact that EVAR is a safe procedure, even though patients became older during the study period. Although the anatomy of patients undergoing OSR has become more challenging, careful patient selection allows OSR to be performed without an elevated risk [25,26]. An observational study from the US in 2020 highlighted the increased anatomical complexity of OSR cases in a tertiary care centre without raising the hospital mortality risk [27]. In contrast to the findings from registry data [5,28], we were unable to demonstrate any time-related trends for demographic characteristics such as age, maximum aneurysm diameter or gender for EVAR and OSR.

Furthermore, we observed changes in procedural aspects. The overall procedure time and percentage of cases with suprarenal clamping significantly increased for OSR. Recent data from a tertiary centre supports our findings. The data indicates that suprarenal clamping has been used more frequently during open repair in the last decade. With a median implementation rate of around 29%, the regression analysis also presented an upward trend in this manner [29]. The increased use of EVAR as standard treatment—leaving more complex cases with shorter or hostile necks to be treated with OSR—could explain the longer procedure time and the higher overall hospital mortality.

Registry data from the US in 2022 suggests an association between higher hospital mortality rates and the growing complexity of cases treated with OSR over the past decade. Similarly, an association of higher cardiac complications and blood transfusions after OSR with increasing mortality was observed [30]. The use of proximal clamping represents a higher burden for the cardiovascular system, which could explain the higher mortality association. A registry study from the US in 2017 presented results similar to ours, even though no analysis of time difference was conducted [19]. A decline in procedure time was also noticed in the EVAR group, which might be traced back to growing expertise with endovascular procedures and increased use of percutaneous access. Supporting these findings, a contemporary review identified a correlation between a significant reduction of around 15–25% in procedure time and a total percutaneous access for EVAR [31].

Moreover, our data detected increased implementation of fenestrated or branched endografts. Recent data from a meta-analysis presented an increase of up to 23% in the use of complex EVAR over the past decade [32]. This might result from a growing reluctance to treat AAAs with short necks with standard EVAR due to long-term durability concerns. Although the use of EVAR outside the instructions for use without increasing hospital mortality has been previously reviewed [33], the increased implementation of fenestrated and branched devices has proven to be a reliable alternative for complex anatomy without increasing the mortality risk compared to OSR [34,35]. Therefore, complex endografts are increasingly accepted as the standard of care for AAAs with hostile necks or juxtarenal AAAs.

The present study showed a lower survival rate for patients undergoing EVAR than for those treated with OSR. In contrast, the 15-year follow-up data of the EVAR-1 trial showed lower mortality rates for patients in the EVAR group up to 8 years after the operation. Thereafter, patients in the OSR group had lower mortality [13]. A reason for the discrepancy in results might be that patients were not randomized in the present study. Instead, the treatment modality was selected based on the patient’s age, comorbidities and frailty. Consequently, patients in the OSR group were younger and exhibited superior overall health status. When interpreting survival differences after EVAR and OSR, the higher age of EVAR patients at the time of the index procedure and the associated lower natural life expectancy must be considered.

Others refer to cardiovascular-related death and malignant disease as common causes of death during follow-up for up to 12 years, without significant differences between survival after EVAR and OSR [14]. Most causes of death during follow-up in our cohort were unknown. However, the limited number of documented causes included respiratory insufficiency, cancer, and multiorgan failure. The low percentage of aneurysm-related deaths in our cohort might be explained by a lack of patient adherence to follow-up visits, since aneurysm-related deaths might remain undetected. A multicenter study from the UK in 2019 suggested an association between follow-up non-compliance and higher mortality during surveillance due to undetected aneurysm-related deaths [36]. Recent registry data has identified cancer as an increasing cause of death in iAAA patients after EVAR during follow-up [21]. There is a crucial need for routine clinical and imaging follow-up, along with patients’ adherence to follow-up algorithms. This adherence is vital for improving our understanding of long-term outcomes when comparing EVAR to OSR.

## 5. Limitations

Our single-centre study is subject to various limitations. First, as no randomization was performed, confounding is likely. As patients were treated with the respective surgical techniques for distinct reasons, confounding by indication is present. Although the study was conducted at an aortic centre with a high patient volume, caution is warranted against generalizing these results. Since associations and correlations were observed, the results may not be transferable to other healthcare systems with a different patient population and surgical expertise. The study design is not appropriate for comparing EVAR to OSR but rather for describing treatment trends and outcomes in a large aortic centre.

As a retrospective analysis was conducted, this study depends on the accuracy of data registration and may be subject to entry errors. However, data were entered on time, with a relatively low risk of error. Nevertheless, especially follow-up data were entered retrospectively. Difficulties regarding telephone contact with patients and their primary care doctors comprise one reason for the loss to follow-up. Furthermore, the definition of iAAA, including juxtarenal aneurysms, could be more precise. Juxtarenal aneurysms often present complex and hostile anatomies, which cannot be treated with a standard EVAR device. For this reason, stent graft technologies have changed over time. In the future, a stricter treatment comparison between infrarenal and juxtarenal aneurysms separately would be reasonable.

Despite these limitations, the current study presents an accurate overview of 18 years of experience treating iAAA in a large German aortic centre. As consecutive patients were included, selection bias is low, and the results are to be interpreted as a precise reflection of contemporary everyday practice.

## 6. Conclusions

This single-centre study demonstrates lower in-hospital mortality for patients treated with EVAR. Despite increasing patient age in the EVAR group and more complex cases in the OSR group, mortalities after both treatment modalities remained stable over the 18-year period. However, after discharge, patients treated with OSR exhibited prolonged survival compared to those treated with EVAR. These findings underscore the importance of careful patient selection for maintaining stable mortality rates, even in an aging and increasingly comorbid population.

## Figures and Tables

**Table 1:** Baseline characteristics.

	Overall	OSR	EVAR	*p*-Value
	**(*n* = 1237)**	**(*n* = 374)**	**(*n* = 863)**	
**Demographics**				
Age in years (mean; SD)	72 (8.1)	68 (7.6)	74 (7.7)	<0.001
Age in years (median; Q1–Q3)	73 (67–78)	69 (64–74)	75 (69–79)	<0.001
Male sex	1096 (89)	328 (88)	768 (89)	0.51
**AAA characteristics**				
Aneurysm diameter in mm (mean; SD)	57 (10)	59 (12)	57 (9.9)	0.14
Aneurysm diameter in mm (median; Q1–Q3)	56 (52–61)	56 (52–62)	56 (52–60)	0.33
Infrarenal location	1047 (85)	285 (76)	762 (88)	<0.001
Juxtarenal location	109 (15)	89 (24)	101 (12)	<0.001
Aneurysmatic iliac arteries	120 (9.7)	56 (15)	64 (7.4)	<0.001
**Comorbidities**				
Hypertension	1059 (86)	300 (80)	759 (88)	<0.001
Smoking	915 (74)	294 (79)	621 (72)	0.014
PAD	187 (15)	62 (17)	125 (15)	0.35
CAD	538 (44)	137 (37)	401 (47)	0.001
Hyperlipidaemia	750 (61)	239 (64)	511 (59)	0.12
Diabetes	233 (19)	57 (15)	176 (20)	0.033
COPD	239 (19)	86 (23)	153 (18)	0.031
Renal insufficiency	216 (18)	81 (22)	135 (16)	0.010
BMI ≥ 30 kgm^−2^	249 (20)	61 (16)	188 (22)	0.29
BMI (mean ± SD)	27 ± 4.2	26 ± 4.1	27 ± 4.3	0.034
Myocardial infarction	220 (18)	42 (11)	178 (21)	<0.001
History of PTCA	359 (29)	63 (17)	296 (34)	<0.001
Atrial fibrillation	190 (15)	26 (6.9)	164 (19)	<0.001
Active malignancy/cancer treatment in the past 10 years	204 (16)	37 (9.8)	167 (19)	<0.001
**ASA classification**				
I	7 (0.57)	5 (1.34)	2 (0.23)	
II	432 (35)	149 (40)	283 (33)	
III	745 (60)	213 (57)	534 (62)	0.005
IV	37 (3.0)	7 (1.87)	31 (3.5)	

Data are given as *n* (%) unless otherwise stated. Categorical data are presented as absolute numbers and percentages; numerically scaled data are presented as median with 1st–3rd quartiles (Q1–Q3), as the data do not follow normality. The Chi-squared and Fisher’s *t*-test were used to determine differences in nominally and interval-scaled data between EVAR and OSR. Mood’s median test was used to determine the differences between numeric data. OSR = open surgical repair, EVAR = endovascular aortic repair, SD = standard deviation, PAD = peripheral artery disease, CAD = coronary artery disease, COPD = chronic obstructive pulmonary disease, BMI = body mass index, PTCA = percutaneous transluminal coronary angioplasty ASA = American Society of Anesthesiologists.

**Table 2:** In-hospital outcomes.

	Overall	OSR	EVAR	*p*-Value
	**(*n* = 1237)**	**(*n* = 374)**	**(*n* = 863)**	
**Length of stay (median; Q1–Q3)**				
Hospital stay (days)	8 (6–11)	10 (8–14)	7 (5–9)	<0.001
ICU stay (days)	0 (0–1)	1 (1–3)	0 (0–1)	<0.001
**In-hospital mortality**	21 (1.70)	13 (3.48)	8 (0.93)	0.001
**Surgical complications**	155 (12.5)	61 (16.3)	94 (10.9)	0.008
**Major**	111 (9.30)	45 (12.6)	66 (7.90)	0.011
IIIa		1	6	
IIIb		44	60	
**Minor**	44 (3.91)	16 (4.86)	28 (3.51)	0.29
IIIb		16	28	
Medical complications *	222 (17.9)	134 (35.8)	88 (10.2)	<0.001
**Multiorgan failure (IVb)**		9	8	
**Respiratory**	56 (5.82)	40 (10.69)	16 (1.85)	<0.001
I		3	-	
II		25	9	
IIIa		3	4	
IVa		9	3	
**Renal**	90 (7.52)	53 (14.2)	37 (4.29)	<0.001
I		12	6	
II		19	21	
IIIa		3	1	
IVa		19	4	
**Cardiovascular**	53 (4.61)	29 (7.75)	24 (2.78)	0.001
II		12	6	
IIIa		14	17	
IVa		3	1	
**Gastrointestinal**	25 (2.43)	21 (5.61)	4 (0.46)	<0.001
I		3	-	
II		11	3	
IIIa		7	1	
**Neurological**	12 (1.13)	6 (1.60)	6 (0.69)	0.027
IVa		6	6	

Data are given as *n* (%) unless otherwise stated. The chi-squared test was used to determine differences in nominally scaled data between EVAR and OSR. Causes of death as well as major and minor complications are listed in the Supplemental Materials. A Mood median equality test for length of stay was conducted. Q1 = first quartile, Q3 = third quartile, ICU = intensive care unit. * = Number of patients with a medical complication. Surgical complications were classified as minor (i.e., wound infection, wound dehiscence, wound hematoma, lymphocele, pseudoaneurysm of the access artery) and major complications (i.e., severe bleeding; acute limb ischemia; acute stent graft thrombosis, occlusion, stenosis, or perforation of the artery; visceral ischemia; visceral complications; rupture; surgical site infection; graft infection; device disruption; abdominal compartment syndrome). Medical complications included respiratory (i.e., respiratory insufficiency with long-term mechanical ventilation, pneumonia, severe pleural effusion, pneumothorax), renal (i.e., acute renal insufficiency, urosepsis due to severe urinary tract infection), cardiac (i.e., myocardial infarction, acute heart failure, hemodynamically relevant arrhythmias), gastrointestinal (i.e., paralytic ileus, acute pancreatitis, ischemic colitis) and neurological (subarachnoid bleeding, spinal ischemia, stroke) complications. Surgical and medical complications are categorized according to Clavien–Dindo. In some cases, patients had more than one medical complication.

**Table 3:** Temporal trends for EVAR.

	Total *n* = 863	2005–2010 *n* = 205	2011–2016 *n* = 363	2017–2022 *n* = 295	∆ per Year	*p*-Value
**Demographics**						
AAA diameter in mm (mean; SD)	57.5 (9.62)	58.4 (10.2)	57.0 (9.77)	57.5 (9.00)	−0.05	0.44
Age in years (mean; SD)	74.0 (7.72)	73.3 (7.66)	73.9 (7.51)	74.4 (8.02)	0.08	0.17
Male sex	768 (89.0)	191 (93.2)	312 (86.0)	265 (89.8)	−0.02	0.52
**Length of stay (median; Q1–Q3)**						
Hospital stay (days)	7 (5–9)	7 (6–11)	7 (6–9.5)	6 (4–8)	−0.02	<0.001
ICU stay (days)	0 (0–1)	1 (0–1)	0 (0–0)	0 (0–0)	−0.06	0.004
**In-hospital mortality**	8 (0.93)	1 (0.49)	3(0.83)	4 (1.36)	0.08	0.35
**Complications**						
Surgical	94 (10.9)	17 (8.29)	52 (14.3)	25 (8.47)	−0.003	0.89
Medical *	88 (10.2)	16 (7.80)	36 (9.92)	36 (12.2)	0.04	0.094
**Intraoperative Characteristics**						
Procedure time in minutes (median)	123	132	127	110	−1.97	0.002
Percutaneous access	382 (44.3)	0 (0.00)	126 (34.7)	256 (86.8)	0.64	<0.001
Uni-iliac device	21 (2.43)	8 (3.90)	9 (2.48)	4 (1.36)	−0.07	0.13
Fenestrated or branched device	73 (8.46)	4 (1.95)	30 (8.26)	39 (13.2)	0.15	<0.001

Data are given as *n* (%) unless otherwise stated. Temporal trends were analysed using linear and logistic regression modelling. Reference group: 2005–2010; *n* = 205. The results are presented as the difference per year (∆ per year). EVAR = endovascular aneurysm repair, AAA = abdominal aortic aneurysm, SD = standard deviation, Q1 = first quartile, Q3 = third quartile, ICU = intensive care unit. Length of stay (Poisson regression). ICU stay (negative binomial regression). * = Number of patients with a medical complication.

**Table 4:** Temporal trends for OSR.

	Total *n* = 374	2005–2010 *n* = 158	2011–2016 *n* = 105	2017–2022 *n* = 111	∆ per Year	*p*-Value
**Demographics**						
AAA diameter in mm (mean; SD)	58.5 (11.8)	58.3 (10.0)	58.6 (12.2)	58.8 (14.6)	−0.003	0.98
Age in years (mean; SD)	68.4 (7.62)	68.7 (6.93)	66.6 (8.32)	69.6 (7.62)	0.04	0.61
Male sex	328 (87.7)	145 (91.8)	86 (81.9)	97 (87.4)	−0.04	0.18
**Length of stay (median; Q1–Q3)**						
Hospital stay (days)	10 (8–14)	9 (8–12)	11 (8–14)	11 (8–16.5)	0.02	<0.001
ICU stay (days)	1 (1–3)	2 (1–3)	1 (1–2)	1 (1–2)	−0.01	0.25
**In-hospital mortality**	13 (3.48)	5 (3.16)	4 (3.81)	4 (3.60)	0.01	0.79
**Complications**						
Surgical	61 (16.3)	21 (13.3)	21 (20.0)	19 (17.1)	0.04	0.17
Medical *	134 (35.8)	49 (31.0)	41 (39.0)	44 (39.6)	0.02	0.34
**Intraoperative Characteristics**						
Procedure time in minutes (median)	210	196	235	216	3.29	<0.001
EA of the aorta, iliac arteries or femoral arteries	207 (55.3)	75 (47.5)	61 (58.1)	71 (64.0)	0.03	0.12
Suprarenal clamping	61 (16.3)	17 (10.8)	22 (21.0)	22 (19.8)	0.06	0.037
Left retroperitoneal access	217 (58.0)	75 (47.5)	61 (58.1)	81 (73.0)	0.10	<0.001

Data are given as *n* (%) unless otherwise stated. Temporal trends were analysed using linear and logistic regression modelling. Reference group 2005–2010; *n* = 205. The results are presented as the difference per year (∆ per year). OSR = open surgical repair, AAA = abdominal aortic aneurysm, SD = standard deviation, Q1 = first quartile, Q3 = third quartile, ICU = intensive care unit, EA = endarterectomy. Length of stay (Poisson regression). ICU stay (negative binomial regression). * = Number of patients with a medical complication.

**Table 5:** Univariate analysis: associations with in-hospital mortality.

		%	OR	CI	*p*-Value
**Demographics**					
Age > median	No	1.45	Ref.	Ref.	
	Yes	1.94	1.34	[0.56–3.33]	0.51
Male sex	No	2.84	Ref.	Ref.	
	Yes	1.55	0.52	[0.19–1.89]	0.29
**Anatomic characteristics**					
AAA diameter > median	No	1.15	Ref.	Ref.	
	Yes	2.40	2.09	[0.87–5.39]	0.092
Infrarenal AAA	No	7.37	Ref.	Ref.	
	Yes	0.67	0.09	[0.03–0.21]	<0.001
**Comorbidities**					
Hypertension	No	1.69	Ref.	Ref.	
	Yes	1.70	0.97	[0.32–4.34]	1.00
Smoking	No	1.55	Ref.	Ref.	
	Yes	1.75	1.10	[0.42–3.47]	0.82
PAD	No	1.43	Ref.	Ref.	
	Yes	3.21	2.32	[0.81–5.85]	0.12
CAD	No	1.86	Ref.	Ref.	
	Yes	1.49	0.80	[0.31–1.94]	0.62
Hyperlipidaemia	No	2.05	Ref.	Ref.	
	Yes	1.47	0.71	[0.29–1.73]	0.44
Diabetes	No	1.49	Ref.	Ref.	
	Yes	2.58	1.77	[0.61–4.45]	0.26
COPD	No	1.10	Ref.	Ref.	
	Yes	4.18	3.92	[1.60–9.50]	0.003
Renal insufficiency	No	1.57	Ref.	Ref.	
	Yes	2.31	1.52	[0.48–3.97]	0.39
Obesity BMI ≥ 30 kgm^−2^	No	1.33	Ref.	Ref.	
	Yes	2.41	1.85	[0.62–4.98]	0.25
Myocardial infarction	No	1.57	Ref.	Ref.	
	Yes	2.27	1.49	[0.47–3.88]	0.40
PTCA	No	1.71	Ref.	Ref.	
	Yes	1.67	0.99	[0.35–2.49]	0.96
Atrial fibrillation	No	1.81	Ref.	Ref.	
	Yes	1.05	0.62	[0.09–2.16]	0.76
Active malignancy/cancer treatment in the past 10 years	No	1.55	Ref.	Ref.	
	Yes	2.45	1.64	[0.52–4.28]	0.37
ASA classification	I/II	1.37	0.93	[0.34–2.52]	0.88
	III	1.48	Ref.	Ref.	
	IV	10.0	7.87	[2.38–26.0]	0.001
**Procedural aspects**					
OSR			Ref.	Ref.	
EVAR			0.26	[0.10–0.63]	0.001

PAD = peripheral artery disease, CAD = coronary artery disease, COPD = chronic obstructive pulmonary disease, PTCA = percutaneous transluminal coronary angioplasty, BMI = body mass index, ASA = American Society of Anesthesiologists, OR = odds ratio, CI = confidence Interval.

## Data Availability

The data that underpin these findings are not freely accessible due to restrictions on disclosure imposed by the need to protect the sensitivity of the information. Upon reasonable request, data are available from the corresponding author.

## Supplementary Materials

The following supporting information can be downloaded at https://www.mdpi.com/article/10.3390/jcm15041353/s1: Table S1: Surgical characteristics for OSR, Table S2: Surgical characteristics for EVAR, Table S3: Causes of in-hospital death, Table S4: Surgical complications, Table S5: Causes of death during follow-up, Table S6: Follow-up; Figure S1: Normal distribution of patients’ survival data, Figure S2: Cox proportional regression model for cumulative survival probability 5 years after the index procedure regarding different preoperative and treatment characteristics for the intact abdominal aneurysm cohort.
