# Supplementary material for: Endovascular and Open Surgical Repair of Intact Abdominal Aortic Aneurysms: Outcomes, Treatment Trends and Early Mortality Risk Factors in a Single-Centre Study over 18 Years
Source: J Clin Med. 2026 Feb 9;15(4):1353. doi: 10.3390/jcm15041353 (PMC12941369; doi:10.3390/jcm15041353)
Supplement: Supplementary file 1 [file jcm-15-01353-s001.zip › jcm-3968536-supplementary.pdf]

SUPPLEMENTARY MATERIAL

Tables

Supplementary Table S1: Surgical characteristics for OSR

**Surgical access *n* (%)**	
**Retroperitoneal**	217 (58.0)
**Median laparotomy**	157 (42.0)
**Surgery time in minutes (median; range)**	210 (166-270)
**Reconstruction type**	
**Tube**	226 (60.4)
**Aorto-biiliacal**	144 (38.5)
**Aorto-bifemoral**	2 (0.53)
**Unsuccessful graft implantation or patient death during operation**	2 (0.53)
**Endarterectomy of the aorta, iliac arteries or femoral arteries**	207 (55.3)
**Reinsertion of ≥ 1 artery**	36 (9.63)
**Ligation of the inferior mesenteric artery**	202 (54.0)
**Suture ligation of lumbar arteries**	317 (84.8)
**≥ 1 lumbar artery**	169 (45.2)
**≥ 3 lumbar arteries**	148 (39.6)
**Clamping *n, (%)***	
**Infrarenal**	273 (73.0)
**Suprarenal**	61 (16.3)
**Interrenal**	13 (3.48)
**Supramesenteric**	8 (2.14)
**Supraceliac**	17 (4.55)
**Cold kidney perfusion**	44 (11.8)

Data are given as *n* (%) unless otherwise stated. OSR= open surgical repair.

Supplementary Table S2: Surgical characteristics for EVAR	
**Surgical access *n* (%)**	
**Cutdown**	481 (55.6)
**Percutaneous**	382 (44.3)
**Procedure time in minutes (median; range)**	123 (97- 172)
**Extent of EVAR**	
**Tube**	21 (2.43)
**Aorto-biiliacal**	830 (96.2)
**Aorto-monoiliacal**	12 (1.39)
**Device type and manufacturer**	
**Endurant II (Medtronic)**	281 (32.6)
**Talent (Medtronic)**	10 (1.16)
**Zenith Alpha (Cook)**	55 (6.37)
**T-Branch (Cook)**	8 (0.93)
**Zenith Flex (Cook)**	157 (18.2)
**Excluder (Gore)**	60 (7.00)
**Treo, Treovance (Bolton Medical, Terumo Aortic)**	77 (8.92)
**Anaconda (Vascutec, Terumo Aortic)**	57 (6.60)
**Incraft (Cordis)**	35 (4.06)
**CMD (Cook, Vascutec, Terumo Aortic)**	64 (7.42)
**PhMD**	1 (0.12)
**Endoleak at discharge**	237 (27.5)
**Ia**	45 (5.21)
**Ib**	6 (0.69)
**II**	164 (19.0)
**III**	19 (2.20)
**Mixed**	3 (0.35)

Data are given as n (%) unless otherwise stated. EVAR= endovascular aortic repair. Medtronic (Parkway, Minneapolis, MN 55432-5604 USA); COOK MEDICAL (LLC 750 Daniels Way, P.O. Box 489 Bloomington, IN 47402-0489 USA); Terumo (Terumo Europe NV, Interleuvenlaan 40, 3001 Leuven, Belgium); Gore (W. L. Gore & Associates, 555 Paper Mill Road, Newark, DE 19711, USA); Cordis (Cordis Germany GmbH Südportal 1, 22848 Norderstedt, Germany).

**Supplementary Table S3: Causes of in-hospital death**	**OSR (*n*=12)**	**EVAR (*n*= 8)**
**Cardiovascular failure or acute myocardial infarction with acute heart failure and cardiogenic shock**	4	2
**Sepsis with multiorgan failure**	2	1
**Bleeding with haemorrhagic shock**	4	1
**Multiorgan failure**	1	3
**Stroke due to intracerebral haemorrhage**	-	1
**Ventricular fibrillation or other arrhythmia causing cardiogenic shock**	1	-

**Supplementary Table S4: Surgical complications**	**OSR**	**EVAR**
Major	*n*=45	*n*=66
**Severe bleeding**	21	11
**Acute limb ischemia**	8	21
**Acute stent graft thrombosis**	-	23
**Perforation of the artery**	-	3
**Visceral complications***	15	14
**Aneurysm rupture**	1	-
**Surgical site infection**	-	3
**Device disruption**	-	1
Minor	*n*=16	*n*=29
**Wound dehiscence**	15	27
**Wound hematoma**	1	-
**Aneurysm of the access artery**	-	2

*Including visceral ischemia, abdominal compartment syndrome, EVAR= endovascular aortic repair, OSR= open surgical repair.

**Supplementary Table S5: Causes of death during follow-up**	**OSR (*n*=10)**	**EVAR (*n*=63)**
**Unknown**	2	47
**Pulmonal (ARDS after COVID, respiratory insufficiency)**	1	1
**Sepsis with multiorgan failure**	1	3
**Haemorrhagic shock after aneurysm sac rupture due to endoleaks**	-	2
**Cardiovascular failure due to acute myocardial infarction or cardiogenic shock**	2	6
**Brain hypoxia after aspiration**	1	-
**Cancer**	2	4
**Haemorrhagic shock after bleeding due to rupture of the Anastomosis**	1	-

EVAR= endovascular aortic repair, OSR= open surgical repair.

Supplementary Table S6: Follow-up	OSR	EVAR
**Median follow-up in months (Q1–Q3)**	17 (1–53)	21 (3–54)
**Loss of follow-up after discharge***	63	117
**Contrast-enhanced ultrasound**		333
**CT angiography**	51	281
**Ultrasound**	247	131
**Clinical follow-up**	-	1

*= Number of patients who were lost to follow-up immediately after discharge, without any kind of follow-Up. EVAR= endovascular aortic repair, OSR= open surgical repair.

Figures

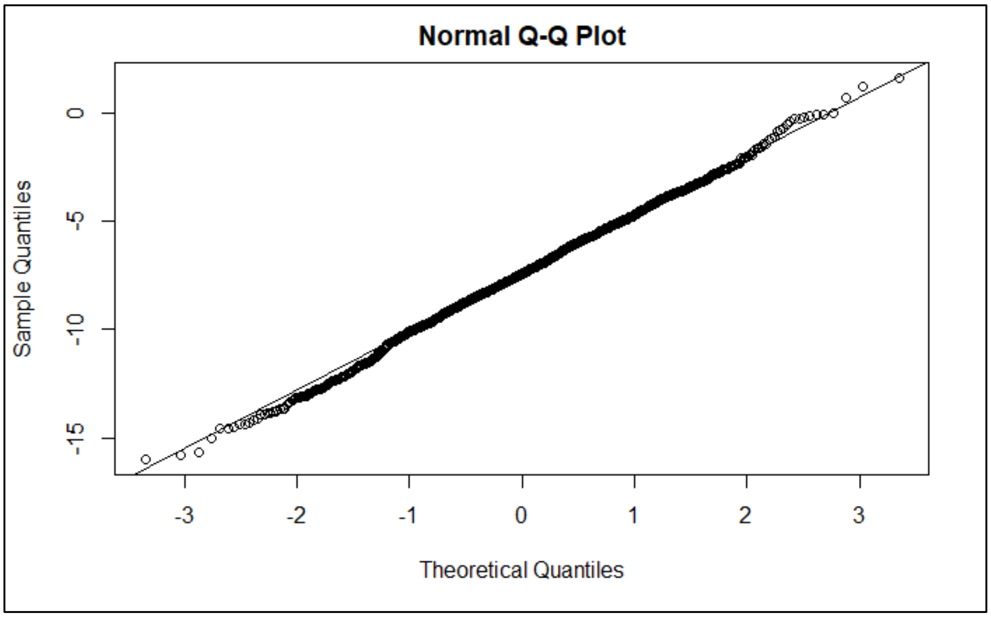

Supplementary Figure S1: Normal distribution of patients' survival data.

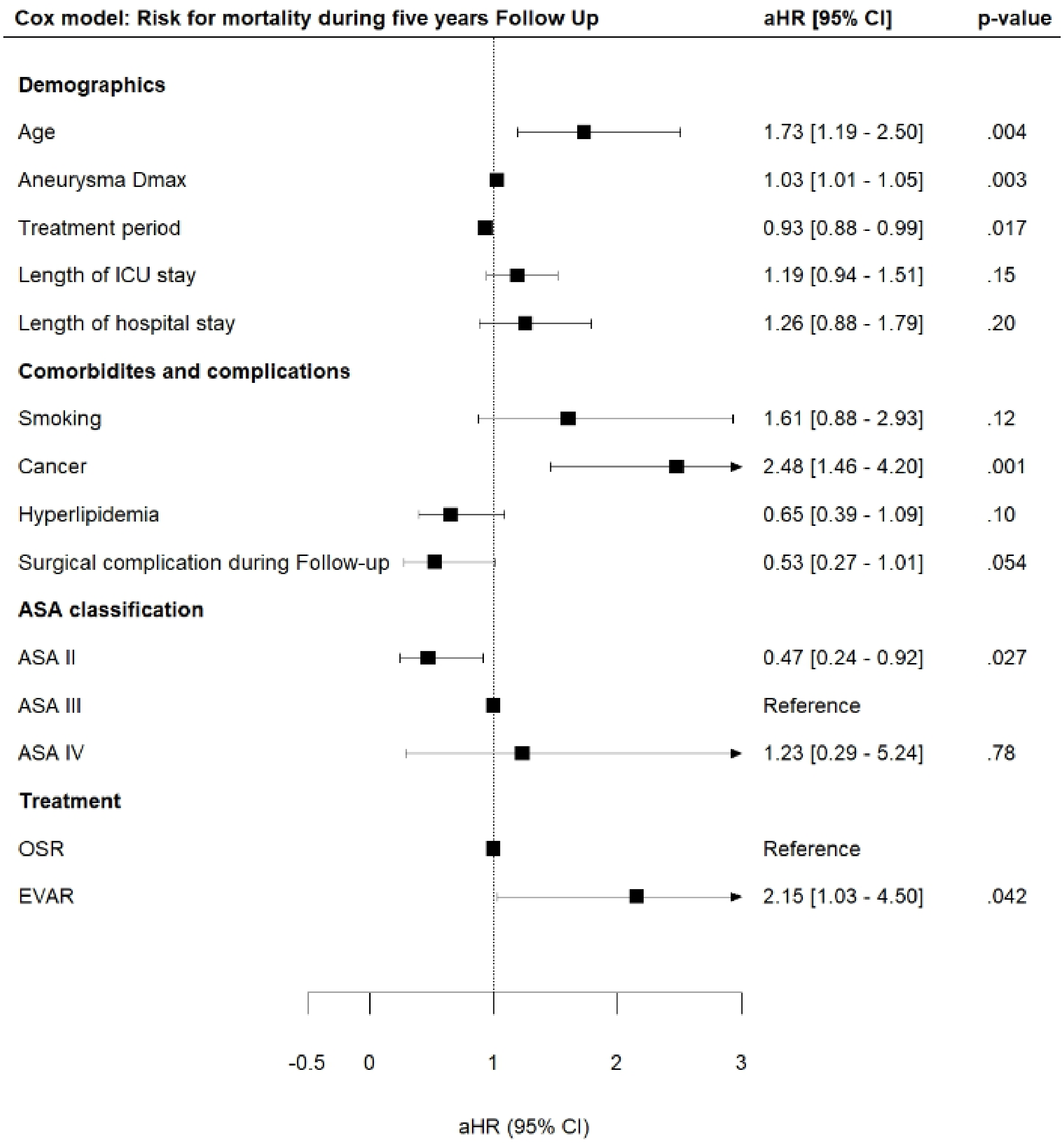

Supplementary Figure S2: Cox proportional regression model for cumulative survival probability 5 years after the index procedure regarding different preoperative and treatment characteristics for the intact abdominal aneurysm cohort. aHR= adjusted hazard ratio, CI= confidence interval, Dmax = maximal aneurysm diameter, PAD= peripheral artery disease, CAD= coronary artery disease, COPD=chronic obstructive disease, ASA= American Society of Anesthesiologists, Length of hospital stay= log2 transformation, length of ICU stay=square root transformation.
